# Prevalence of gender-affirming hormone therapy in non-binary and genderqueer individuals, a systematic review and meta-analysis

**DOI:** 10.1007/s12020-025-04381-x

**Published:** 2025-08-13

**Authors:** Jade M. Castelijn, Bodi Huisman, Thomas D. Steensma, S. Annelijn Wensing-Kruger, Baudewijntje P. C. Kreukels, Martin den Heijer, Koen M. A. Dreijerink

**Affiliations:** Amsterdam UMC, location VU University, Center of Expertise on Gender Dysphoria, Amsterdam, The Netherlands

**Keywords:** Gender-affirming hormone therapy, Non-binary genderqueer, NBGQ, Transgender

## Abstract

**Purpose:**

Current guidelines for gender-affirming hormone therapy (GAHT) primarily focus on binary transgender (BT) individuals and provide limited recommendations for non-binary and genderqueer (NBGQ) individuals. Understanding hormone use among this heterogenous group will contribute to more personalized counseling and treatment strategies. We performed a systematic review of the scientific literature to assess the prevalence of GAHT in NBGQ individuals and potential clinical context-dependent differences.

**Methods:**

A systematic literature search was performed aimed to assess the prevalence and type of GAHT use in NBGQ and BT individuals according to Preferred Reporting Items for Systematic Reviews and Meta-analysis (PRISMA) guidelines, using PubMed, Embase and Web of Science databases.

**Results:**

Sixteen eligible articles were identified. All were retrospective cohort series published between 2018–2024, including a total of 1948 NBGQ individuals and 3991 BT individuals. Hormone use varied from 4–93% in NBGQ individuals and from 52–95% among BT individuals. Overall, significantly fewer NBGQ individuals were on GAHT compared to BT individuals, except those referred to gender-affirming care clinics. GAHT was more frequent in clinical cohorts compared with non-clinical cohorts, both among NBGQ (OR 6.4; CI 5.1–8.0) and BT (OR 3.1; CI 2.6–3.8) individuals. There was insufficient Information in the literature to be able to draw conclusions with regard to differences in types of GAHT.

**Conclusion:**

The systematic review confirms that GAHT is less common in NBGQ compared with BT individuals. Hormone use is more frequent among NBGQ individuals seeking care in a clinical setting. These results highlight the heterogeneity in NBGQ as well as BT individuals with regard to treatment needs. Caregivers, in particular in clinical settings, should be aware that not all NBGQ individuals seek GAHT. Additional studies are needed to further explore tailored endocrine treatment needs in NBGQ individuals.

## Introduction

Many individuals experience their gender in ways that extend beyond a binary framework. The umbrella term non-binary genderqueer (NBGQ) is often used in Western contexts as a collective term for people with such gender identities [1]. The term includes gender diverse or gender nonconforming experiences, such as identifying as neither male nor female, identifying with multiple genders (e.g., bigender, polygender), experiencing a gender that changes over time (e.g., gender fluid), or identifying with no gender (e.g., agender) [2, 3]. These labels may hold different meanings across cultural contexts and can change over time and in different settings. The prevalence of non-binary and genderqueer (NBGQ) individuals varies widely depending on the population studied and the methods used to assess gender identity. While the availability of comprehensive data remains limited, recent studies estimate that 0.3–4.6% of individuals in the general population self-identify as NBGQ [4–7]. In transgender and gender-diverse populations, this prevalence is substantially higher, ranging from 18.5–50% [8, 9]. In the 2022 U.S. Transgender Survey, 38% of respondents identified as non-binary [10]. Thus, there appears to be variability in prevalence based on context.

Some NBGQ individuals seek gender-affirming medical therapy (GAMT), while others do not [2]. In recent years, increasingly more NBGQ individuals seek GAMT [11]. For those who seek care, gender-affirming hormone therapy (GAHT) and surgery are among the options available. In the case of binary transgender (BT) individuals, conventional GAHT to feminize consists of estradiol and anti-androgenic treatment for persons assigned male sex at birth (AMAB) and testosterone to masculinize for persons who were assigned female sex at birth (AFAB) [2, 12]. While most NBGQ individuals who choose GAHT may opt for similar treatments as BT individuals, recent studies suggest there may be a greater need for tailored approaches within this group. These may include options such as micro dosing or short-term hormone use [13, 14]. However, there are no specific recommendations in endocrinology guidelines for NBGQ persons other than to consider tailored approaches [2]. Further insight into NBGQ GAHT needs, possibly context-dependent, will inform future guidelines for counseling and treatment. In order to assess the need for GAHT in the NBGQ population, we performed a systematic review of the prevalence and types of GAHT use in NBGQ and BT individuals reported in the scientific literature. We compared clinical with non-clinical/ population-based studies.

## Methods

### Search strategy

This study was registered in PROSPERO (registration number: CRD42020181288). A literature search was performed based on the Preferred Reporting Items for Systematic Reviews and Meta-analysis (PRISMA) statement [15]. To identify all relevant publications on endocrine treatment of NBGQ individuals, we performed systematic searches in the bibliographic databases PubMed, Embase, and the Web of science from inception to February 26, 2024. Search terms included controlled terms as well as free text terms (Supplementary Table).

After removal of duplicates, articles were initially screened by title and abstract to exclude non-relevant reports (JC, KD). Of the remaining articles, full texts were read for eligibility (JC, KD). Any discrepancy regarding article selection was resolved by consensus (JC, KD).

Included were any type of English language original reports addressing GAHT use in NBGQ individuals. Excluded were papers describing participants of <18 years of age, letters to the editor, reviews, editorials and case reports or series including <10 NBGQ subjects.

### Data extraction

The following characteristics were extracted from the included studies: subject characteristics (number of subjects, gender identity), clinical or population-based set-up, country of the study, GAHT use.

### Quality assessment

Study quality and potential bias were assessed independently by two raters (JC, KD). The assessment of studies was conducted using the Critical Appraisal Skills Programme (CASP) [16]. This tool consists of eleven questions, each with response options of “yes”, “no” or “can’t tell”. To assess the risk of bias and methodological quality, the total number of negative responses (i.e., “no” or “can’t tell”) was determined.

### Statistical analysis

For the analysis, data of GAHT were pooled for NBGQ and BT subjects in the various settings: gender-affirming medical care (GAMC) clinics, plastic surgery clinics and population surveys. Weighted means of hormone use were calculated. To compare differences regarding GAHT use between NBGQ and BT individuals, we used Fisher’s exact testing. We assessed odds ratios (+/− 95% confidence intervals) to assess differences between clinical and non-clinical settings.

## Results

**Fig. 1:**
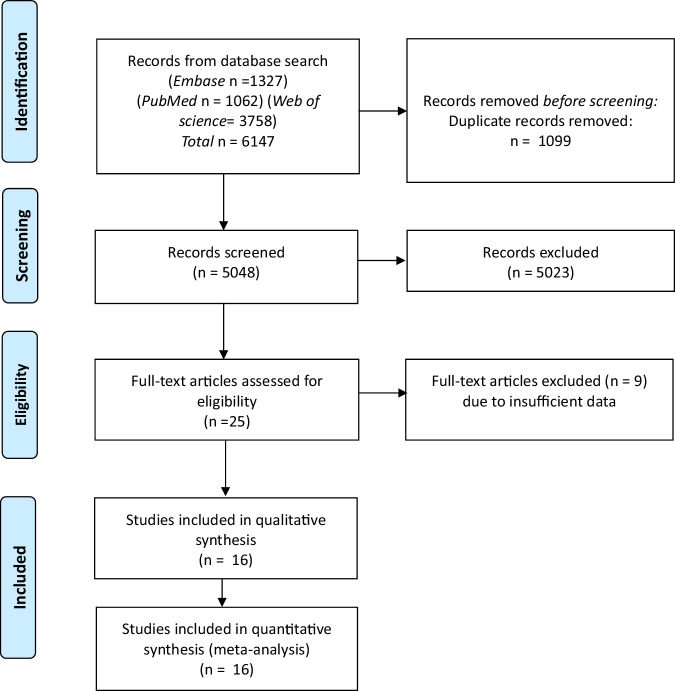
PRISMA flowchart of the literature search and selection process. PRISMA preferred reporting items for systematic reviews and meta-analysis

The initial search resulted in a total of 6147 records: PubMed *n* = 1062, Embase *n* = 1327, Web of science *n* = 3758. Following removal of duplicates (*n* = 1099) and screening all titles and abstracts, a total of 25 papers were subsequently assessed in full text for inclusion, after which 9 were excluded, resulting in a total of 16 studies which could be included for the analysis (Fig. 1) [7, 13, 17–30]. The included studies were determined to meet the threshold for sufficient quality based on the CASP criteria. All 16 studies were retrospective cohort series, published between 2018 and 2024, representing a total of 1948 NBGQ individuals (Table 1). In all but one study, NBGQ identity was assessed through self-reported questionnaires. Hu et al. determined NBGQ identity based on medical records [22]. Thirteen studies also included data on binary transgender individuals in similar contexts (*n* = 3991). Internationally, there is considerable variability in how gender-affirming care is provided. The provider of gender-affirming care may be in a specialized gender clinic, it may be in a primary care setting, or it may be overseen by endocrinologists or plastic surgery departments. In the papers analyzed, eight studies were carried out in a clinical setting: three in clinics providing GAMC [13, 27, 29] and five in plastic surgery clinics [17–19, 22, 26]. In eight studies, participants were recruited via social media and community organizations [7, 20, 21, 23–25, 28, 30]. Nine reports are from the United States, two from the Netherlands and the remaining were from Australia, Italy, Poland, Germany and Korea.

**Table 1:** Ranked list of GAHT (%) in NBGQ individuals (N indicates number of subjects) in 16 selected studies

Study and year	NBGQ GAHT (%)	Total NBGQ n	BT GAHT(%)	Total BT nStudy context	
Fisher et al. [20]	4%	877	52%	624	Population survey
Gawlik et al. [21]	26%	34	67%	129	Population survey
Kennis et al. [7]	26%	62	71%	197	Population survey
Puckett et al. [28]	30%	63	78%	166	Population survey
Barron et al. [17]	31%	13	92%	115	Plastic surgery clinic
Margulies et al. 2018 [24]	32%	68	66%	181	Population survey
McTernan et al. [26]	33%	111	86%	665	Plastic surgery clinic
Cronin et al. 2023 [18]	36%	14	80%	44	Plastic surgery clinic
Koehler et al. [23]	46%	76	77%	339	Population survey
Zwickl et al. [30]	51%	271	NA	NA	Population survey
Roznovjak et al. [29]	59%	17	95%	58	GAMC clinic
Esmonde et al. [19]	64%	58	NA	458	Plastic surgery clinic
Hu et al. [22]	66%	67	NA	NA	Plastic surgery clinic
Marks et al. 2019 [25]	75%	69	93%	682	Population survey
Oh et al. [27]	89%	35	93%	302	GAMC clinic
Van Dijken et al. [13]	93%	113	92%	489	GAMC clinic

In addition, hormone use in BT individuals and the contexts of the studies are shown

*GAHT* gender-affirming hormone therapy, *BT* binary transgender, *NBGQ* non-binary and genderqueer, *GAMC* gender-affirming medical care, *NA* data not available

**Fig. 2:**
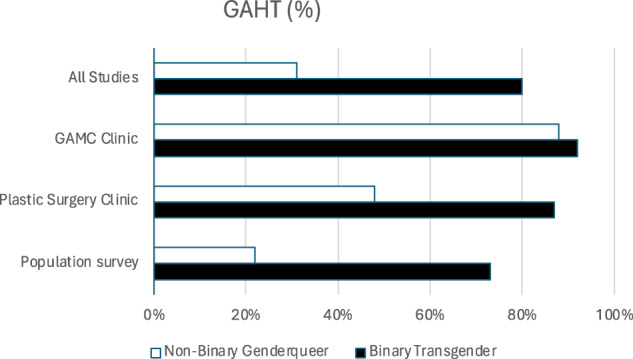
Mean weighted percentages of GAHT use in the different study contexts: overall, gender clinic, plastic surgery and population survey. GAHT gender-affirming hormone therapy, GAMC gender-affirming medical care

GAHT use among NBGQ individuals varied from 4–93% and in BT individuals from 52–95% (Table 1). Across the studies, a mean 31% of NBGQ individuals used hormones versus 80% of BT persons (*p* < 0.0001). The mean prevalence of GAHT in NBGQ and BT individuals in GAMC clinics was 88% vs 92% (*p* < 0.09), in plastic surgery cohorts 48% vs 87% (*p* < 0.0001) and 22% vs 73% (*p* < 0.0001) in population-based online surveys (Fig. 2). In a pooled analysis, GAHT use among NBGQ individuals was higher in subjects in the clinical context versus participants in the non-clinical surveys (OR 6.4; CI 5.1–8.0). To a lesser extent, the same held true for GAHT in BT individuals (OR 3.1; CI 2.6–3.8). The specific type of hormone treatment received was only listed in detail in five studies [13, 21, 22, 25, 30]. These studies reported on the types of GAHT used by NBGQ individuals (e.g., estradiol, testosterone, spironolactone, progesterone) but did not provide detailed information on tailored GAHT or sufficient data to compare hormonal regimens of NBGQ individuals with BT individuals within the same cohorts. The study by Van Dijken et al. was the only one to explicitly mention tailored GAHT and indicated that while overall as many NBGQ as BT individuals did receive GAHT, NBGQ individuals were significantly more likely to receive tailored GAHT, indicating that NBGQ individuals may have different preferences regarding the form of treatment [13].

## Discussion

We performed a systematic review of the scientific literature on the prevalence of GAHT use in NBGQ and BT individuals and found 16 studies that fulfilled the inclusion criteria. The studies were published between 2018–2024, indicating that this is an emerging field in endocrinology. It should also be noted that before the introduction of gender dysphoria as a DSM-5 diagnosis in 2013, non-binary gender identity was not commonly recognized as an indication for treatment. This could also explain, in part, the absence of reports on this topic before 2018.

The studies included a mix of individuals that were assessed in clinical contexts but also in population-based surveys. While all studies reported gender identity, some did not include information on birth-assigned sex, limiting the possibility for further analysis. Also, only five studies included detailed information on the type of GAHT use, and only Van Dijken et al. reported tailored GAHT [13]. While Van Dijken et al. found that NBGQ individuals were more likely to receive tailored GAHT, the other studies did not provide sufficient data to draw conclusions about hormone treatment preferences compared to BT individuals.

In all but one study, NBGQ identity was assessed through self-reported questionnaires. In Hu et al. however, NBGQ identity was determined based on medical records [22]. As mentioned, NBGQ is an umbrella term and therefore the NBGQ cohorts in the various studies may very well represent different populations. There is no certainty that every subject identifies as NBGQ in a similar fashion. In this research field, it is known that various definitions used in the literature affect prevalence estimates and complicate comparisons [31]. From a research perspective, categorizing NBGQ individuals as a distinct group can provide valuable insights into patterns of GAHT use and treatment outcomes. Uniform measuring tools are being developed for this purpose [32]. However, in clinical practice, care must always be personalized, recognizing that NBGQ individuals exist on a broad spectrum of gender identities.

We found that the prevalence of GAHT among NBGQ individuals depends on the context: Of the 16 studies included, eight were non-clinical surveys, for which participants had been recruited via community interest groups and social media. Five studies were conducted in plastic surgery clinics, while three focused on individuals visiting GAMC clinics. GAHT use among NBGQ individuals was higher in subjects visiting clinics (GAMC and plastic surgery) versus participants in the non-clinical surveys. GAHT use in NBGQ individuals was lower compared with GAHT use in BT individuals in both the plastic surgery cohort and the non-clinical cohort, while the prevalence of GAHT use did not significantly differ in the context of the GAMC clinics. These findings are likely due to selection of individuals within the heterogenous NBGQ spectrum with more pronounced treatment desires who apply to gender clinics. However, these observations also imply that within the NBGQ groups many individuals do not use GAHT, whereas in BT individuals GAHT is much more common.

Notably, the reported prevalence of GAHT use varied widely across studies. For example, even among the two studies conducted in breast surgery clinics under seemingly similar circumstances, GAHT use differed nearly twofold (31% vs. 66%) [19, 26]. The particularly low percentage (4%) observed in one study may be partly explained by the way GAHT use was categorized; the survey included multiple response options such as “desire to start GAHT,” “about to start GAHT,” “currently taking GAHT,” and “taken GAHT in the past” [20]. Our study only accounted for current GAHT use, which might have led to an underestimation of the total proportion of individuals with a history of or interest in GAHT.

Of the 16 studies analyzed, only seven reported race/ethnicity in their demographics. Of these seven studies, participant populations consisted of 75.8–92% white individuals. This trend aligns with existing transgender research, which often overrepresents white individuals. For instance, a recent study noted that, despite efforts to recruit a diverse sample, the participants were primarily white [33]. Further, it has been reported that transgender people of color often face greater disparities than their white counterparts across various health and social outcomes [34]. This underscores the need for more inclusive and representative studies that account for intersectionality.

Our observations showed that particularly in non-clinical samples, a substantial number of NBGQ individuals may not seek medical interventions and that GAHT use is significantly lower among NBGQ versus BT individuals outside the clinical setting. Several factors could contribute to this difference. First, research indicates that compared to BT individuals, NBGQ individuals report less body dissatisfaction, and are less likely to pursue gender-affirming medical treatments [11]. Similarly, in a non-clinical cohort, Kennis et al. found that BT individuals report a stronger desire for GAHT than NBGQ individuals [7]. Among those who do not wish to pursue treatment, NBGQ individuals most often refer to their gender identity for not wanting this care, while BT individuals more frequently mention concerns about medical complications [7]. More research is needed in order to assess whether this is less need for GAHT in NBGQ individuals, or whether current GAHT options simply do not fit the needs of NBGQ individuals because current GAHT might be binary in scope.

Beyond personal preferences, barriers to healthcare access may also play a role, which may also be reflected by the 49% of NBGQ individuals who indicated a desire to use hormones in the U.S. Transgender Survey [28, 35]. Financial constraints, lack of knowledgeable providers, but also a lack of fitting medical options are among the many possible barriers NBGQ individuals may face when seeking GAMT. Burchell et al. showed that NBGQ individuals in Canada often face significant obstacles in obtaining gender-affirming care, including the expectation to educate their medical providers about their own needs [36].

This study highlights the lack of research regarding tailored GAHT for NBGQ individuals. As a result, even among those who wish to use hormones, current medical options may feel too binary, making it difficult for some NBGQ individuals to find treatment that aligns with their gender identity.

While those who do not seek medical transition may be less likely to visit a clinic, the insights of this study are highly relevant for healthcare professionals supporting individuals in exploring their gender identity. For endocrinologists and other caregivers in clinical contexts, it is important to recognize that not all NBGQ individuals desire GAHT. NBGQ individuals may require more flexible and individualized approaches rather than conventional, binary treatment models. Given that the needs and preferences of NBGQ individuals can differ from those of BT individuals, a biopsychosocial approach—integrating medical, psychological, and social perspectives—may be valuable in understanding their treatment goals. Interdisciplinary collaboration between endocrinologists, mental healthcare providers, and other healthcare providers could help ensure that care is not only medically appropriate but also aligns with the diverse experiences and identities of NBGQ individuals. Further studies are needed, in particular in large well-defined clinical cohorts, to further determine the endocrine needs of the NBGQ population. This includes monitoring studies that provide insight into GAHT use among NBGQ individuals - whether they pursue GAHT at all and if so in what form - as well as long-term medical outcome studies assessing the effects of tailored interventions. Additionally, qualitative research on the motivations, desires, and perceived outcomes of GAHT in NBGQ individuals is essential to better understand experiences and needs.

## Supplementary information

Supplementary Table

## Data Availability

No datasets were generated or analysed during the current study.
