# Supplementary material for: Prevalence of gender-affirming hormone therapy in non-binary and genderqueer individuals, a systematic review and meta-analysis
Source: Endocrine. 2025 Aug 13;90(2):420–6. doi: 10.1007/s12020-025-04381-x (PMC12572055; doi:10.1007/s12020-025-04381-x)
Supplement: Supplementary file 1 — Supplementary Table [file 12020_2025_4381_MOESM1_ESM.pdf]

Supplementary Table

Pubmed search strategy

((nonbinary [tiab] OR non-binary[tiab] OR genderqueer[tiab] OR NBGQ[tiab] OR gender fluid[tiab] OR gender nonconforming [tiab] OR gender diversity[tiab])) AND (estrogens or testosterone or androgens or hormone or hormones or hormonal or treatment)

Embase search strategy

('non-binary':ti,ab OR nonbinary:ti,ab OR genderqueer:ti,ab OR nbgq:ti,ab OR 'gender fluid':ti,ab OR 'gender nonconforming':ti,ab OR 'gender diversity':ti,ab) AND ('estrogen'/exp OR 'androgen'/exp OR 'testosterone'/exp OR hormone/exp OR hormones/exp OR hormonal/exp OR treatment/exp OR 'therapy'/exp)

Web of science search strategy

#1 (TS=non-binary OR TS=nonbinary OR TS=genderqueer OR TS=NBGQ OR TS=gender nonconforming OR TS=gender fluid OR TS=gender diversity) AND LANGUAGE: (English) AND DOCUMENT TYPES: (Article) Indexes=SCI-EXPANDED, SSCI, A&HCI, ESCI Timespan=All years;

#2 (TS=estrogens OR TS=androgen OR TS=testosterone OT TS=hormones OR TS=hormone OR TS=hormonal OR TS=treatment OR TS=therapy) AND LANGUAGE: (English) AND DOCUMENT TYPES: (Article) Indexes=SCI-EXPANDED, SSCI, A&HCI, ESCI Timespan=All years;

#3 #2 AND#1
